# *Aggregatibacter actinomycetemcomitans*: From the Oral Cavity to the Heart Valves

**DOI:** 10.3390/microorganisms12071451

**Published:** 2024-07-17

**Authors:** Jasminka Talapko, Martina Juzbašić, Tomislav Meštrović, Tatjana Matijević, Dora Mesarić, Darko Katalinić, Suzana Erić, Andrea Milostić-Srb, Josipa Flam, Ivana Škrlec

**Affiliations:** 1 Faculty of Dental Medicine and Health, Josip Juraj Strossmayer University of Osijek, 31000 Osijek, Croatiadkatalinic@fdmz.hr (D.K.);; 2 University Centre Varaždin, University North, 42000 Varaždin, Croatia; 3 Institute for Health Metrics and Evaluation, University of Washington, Seattle, WA 98195, USA; 4 Department for Health Metrics Sciences, School of Medicine, University of Washington, Seattle, WA 98195, USA; 5 Department of Dermatology and Venereology, Clinical Hospital Center Osijek, 31000 Osijek, Croatia; 6 Department of Radiotherapy and Oncology, University Hospital Center Osijek, 31000 Osijek, Croatia; 7 Faculty of Medicine, Josip Juraj Strossmayer University of Osijek, 31000 Osijek, Croatia

**Keywords:** *Aggregatibacter actinomycetemcomitans*, biofilm, disease, oncogenic potential, virulence factors

## Abstract

*Aggregatibacter actinomycetemcomitans* (*A. actinomycetecomitans*) is a Gram-negative bacterial species that is an essential component of the oral microbiota. Due to its aggregative properties, it plays a role in the pathogenesis of human diseases. The presence of the surface proteins Fim, Briae, and microvesicles enables the bacterium to adhere to the epithelial surface and the tooth’s surface. The presence of leukotoxin A (LtxA), which plays an important role in the pathogenicity of the bacterium, has been associated with both periodontitis and the etiology of rheumatoid arthritis (RA). *A. actinomycetecomitans* is also associated with several other systemic diseases and complications, such as endocarditis and different abscesses. In addition to leukotoxin A, *A. actinomycetecomitans* possesses several different virulence factors, including bacteriocins, chemotaxis inhibitory factors, cytotoxic factors, Fc-binding proteins, immunosuppressive factors, lipopolysaccharide collagenase, fibroblast inhibitory factors, antibiotic resistance determinants, adhesins, invasive factors and factors that inhibit the function of polymorphonuclear leukocytes. The ability of *A. actinomycetemcomitans* lipopolysaccharide to induce macrophages to secrete the interleukins IL-1, IL-1β, and tumor necrosis factor (TNF) is of considerable importance. The primary etiologic factor in the pathogenesis of periodontal disease is the oral biofilm colonized by anaerobic bacteria. Among these, *A. actinomycetemcomitans* occupies an important place as a facultative anaerobic bacterium. In addition, *A. actinomycetemcomitans* possesses many virulence factors that contribute to its potential to cause cancer. This article provides an overview of the virulence factors of *A. actinomycetecomitans* and its association with various systemic diseases, its oncogenic potential, and the treatment options for infections caused by *A. actinomycetecomitans*.

## 1. Introduction

*Aggregatibacter actinomycetemcomitans (A. actinomycetemcomitans)* is one of the thousand bacterial species that form the oral microbiota [1]. It was primarily isolated in 1912 from actinomycotic lesions. For a long time, it was considered that it could not be the only causing pathogen of the disease because it was exclusively isolated from actinomycotic lesions [2]. That theory was proved wrong after the first published case report of endocarditis caused by *A. actinomycetemcomitans* in 1964, followed by many studies with compatible findings [3,4]. Although the natural habitat of this bacteria is the oral cavity, today, it is recognized as a pathogen that can cause different infectious diseases—including endocarditis, arthritis, meningitis, osteomyelitis, and pulmonary empyema [5]. In their study, Fan et al. describe an example of a patient with multivalvular endocarditis caused by *A. actinomycetemcomitans* [6]. At the same time, Kulkarni and colleagues point in their study to a connection between the early pathogenesis of rheumatoid arthritis (RA) and *A. actinomycetemcomitans*, which is found in oral bacteria. *A. actinomycetemcomitans* and the virulence factor leukotoxin (LtxA) can significantly accelerate the development of RA in at-risk patients by triggering further citrullination and inflammation [7]. Serious extraoral infections caused by *A. actinomycetemcomitans* include meningitis. However, it is not known whether an active invasive process causes systemic translocation through the epithelial barrier or results from passive leakage into the bloodstream [8]. In addition, the literature describes the case of a 38-year-old male from India with osteomyelitis of the mandible caused by *A. actinomycetemcomitans* [9], while Mesturino and colleagues described the case of a 15-year-old previously healthy boy diagnosed with pulmonary empyema and subphrenic chest wall abscess caused by *A. actinomycetemcomitans* [10]. 

*A. actinomycetemcomitans* is a bacterial species belonging to the genus *Aggregatibacter*, which is an integral part of the family *Pasteurellaceae* [11] (Table 1). Morphologically, *A. actinomycetemcomitans* is a Gram-negative bacillus with a size of 0.4–0.5 µm × 1.0–1.5 µm [12]. In preparations derived from broth cultures or clinical specimens, it can appear as cocci. It shows optimal growth in the presence of 5% CO_2_ [13]. On chocolate agar, colonies have a diameter of less than 0.5 mm after 24 h of incubation; however, after 48 h of incubation, they can reach a diameter of 1–2 mm [5]. 

This facultatively anaerobic oral pathobiont colonizes the oral mucosa at an early age, where it lives as a commensal without causing any harm to the host [14]. Subsequently, *A. actinomycetemcomitans* has the potential to migrate into the gingival crevice, where it can cause an infection that may result in the loss of connective tissue, teeth, and bone supporting tissue [15]. This species is a significant contributor to the development of periodontitis in both adolescents and young adults [16]. Furthermore, *A. actinomycetemcomitans* has been identified as a critical factor in the development of periodontitis and tooth loss in African adolescents. This phenomenon is characterized by familial clustering, which suggests a genetic predisposition to the development of periodontitis [14,17]. Similarly, *A. actinomycetemcomitans* exhibits considerable genetic diversity, with serotypes forming genetically distinct lineages [18]. Genetic diversity leads to the emergence of virulent genotypes, such as JP2 and cagE, as well as harmless genotypes. The common feature of these highly virulent genotypes is increased leukotoxin production [15,19].

To date, seven serotypes have been identified, the differences being due to the immunodominant antigenicity of the O-polysaccharide (O-PS) component of the lipopolysaccharide (LPS) of the outer membrane [20]. The most commonly isolated strains of *A. actinomycetemcomitans* are serotypes a, b, and c. It is important to emphasize that the isolated serotypes mentioned above have different clinical manifestations [21]. More specifically, isolates with serotype b are most commonly identified in cases of severe periodontitis, while isolates with serotypes a and c are typically observed in individuals with either no periodontal disease or mild periodontal disease [22]. The remaining four are less common: d, e, f, and g [12]. Geographical locations may be associated with specific serotypes of *A. actinomycetemcomitans*. For example, the most common serotype in Morocco and India is serotype b, while in Turkey and the UK it is serotype a, and in Finland serotype c [12]. Serotype b is predominant in periodontitis, while serotypes a and c are usually found in healthy individuals [12]. Regarding age, serotype b is more common in people under 35 years of age, and serotype c is more common in people over 35 years of age [23]. In a study of 1449 subgingival plaque samples from Swedish patients with periodontitis, Khzam et al. demonstrated that age significantly influences the prevalence of *A. actinomycetemcomitans*. In particular, they found that the proportion of this bacterium was higher in younger patients than in older patients [24]. This finding is consistent with the National Study on Oral Health of Adults in Australia (2019), which identified age, male gender, lower education, and lack of dental insurance as risk factors for periodontitis [25].

The highly leukotoxic genotypes of *A. actinomycetemcomitans*, JP2 and cagE (serotype b) have been identified as a significant risk factor for the progression of periodontitis in adolescents in North and West Africa [26,27,28,29]. The oral mucosa can also serve as a reservoir for *A. actinomycetemcomitans*, which can subsequently spread and cause systemic infections [30]. In addition to its association with periodontitis, *A. actinomycetemcomitans* has been linked to many other systemic diseases and complications, including (but not limited to) infectious brain abscess, endocarditis, atherosclerosis, chest wall abscess, and many others [14,31,32]. 

*A. actinomycetemcomitans* possesses a variety of virulence factors that trigger a host response possibly related to the pathogenesis of periodontitis [15]. This response allows the bacterium to evade the host’s immune system, which defends the mucosa [8,14]. The primary virulence factor of *A. actinomycetecomitans* is leukotoxin A (LtxA), whose activity is also associated with the presence of autoantibodies, which have been identified as risk markers for rheumatoid arthritis. It has been shown that the occurrence of rheumatoid arthritis correlates with the concentration of anti-LtxA IgM [33]. 

It is important to highlight that *A. actinomycetemcomitans* is a member of the HACEK group (together with *Haemophilus influenze*, *H. parainfluenzae*, *H. aphrophilus*, *H. paraphrophilus*, *Cardiobacterium hominis*, *Eikenella corrodens*, and *Kingella kingae*) of fastidious Gram-negative bacteria [34]. These organisms are part of the normal oral cavity flora and upper respiratory tract. However, they are also capable of causing a variety of infections, including infective endocarditis [34]. The systemic association is not exclusive to the carrier of *A. actinomycetecomitans* but is rather a consequence of the combination of periodontal disease and the prevalence of *A. actinomycetecomitans*. In particular, there is evidence that a reduction in the incidence and prevalence of periodontal disease can lead to a simultaneous reduction in the incidence of associated systemic diseases [35].

The present review will discuss in greater detail the virulence factors of *A. actinomycetemcomitans* that are important in increasing the pathogenicity of this species in both rapidly progressing forms of periodontal disease and in the occurrence of systemic disease. In addition, the clinical manifestations of infections caused by *A. actinomycetemcomitans* and its carcinogenic potential will be addressed thoroughly.

## 2. Data Collection Method

The PubMed database was searched with the following keywords: ‘*Aggregatibacter actinomycetemcomitans*’, ‘biofilm’, ‘oncogenic potential’, and ‘virulence factors’. Additional searches were also carried out on the virulence factors and mechanisms of action of *A. actinomycetemcomitans* in individual organ systems. The search was limited to scientific articles, with the oldest dating back to 1964. In addition, more than half of the articles were published in the last five years. The relevance of the content was determined based on the text of the article. Consequently, the manuscript includes studies conducted in animal models, case reports, case-control studies, cohort studies, comparative studies, meta-analyses, and systematic reviews. A total of 138 related articles were analyzed in this review.

## 3. Virulence Factors of *Aggregatibacter actinomycetecomitans*

*A. actinomycetemcomitans* possesses several virulence factors that contribute to its pathogenicity. These factors enable the bacterium to attach to surfaces, invade tissues, lose the host immune response, and initiate tissue disruption [8]. Table 2 summarizes the important virulence and pathogenicity factors of *A. actinomycetecomitans*.

The adhesins and surface structures (like fimbriae and pili) play crucial roles in their ability to colonize and infect host tissues. These structures allow the bacterium to adhere to host cells and surfaces, resist mechanical removal, and also interact with the host immune system [5]. Fimbriae are thin, hair-like structures assembled from pili, which are about 5 to 7 nanometers in diameter. These pili are made up of a protein known as Flp (fimbrial low-molecular-weight protein), which exhibits a molecular weight of 6.5 kilodaltons (kDa) [5,36]. 

**Figure 1:**
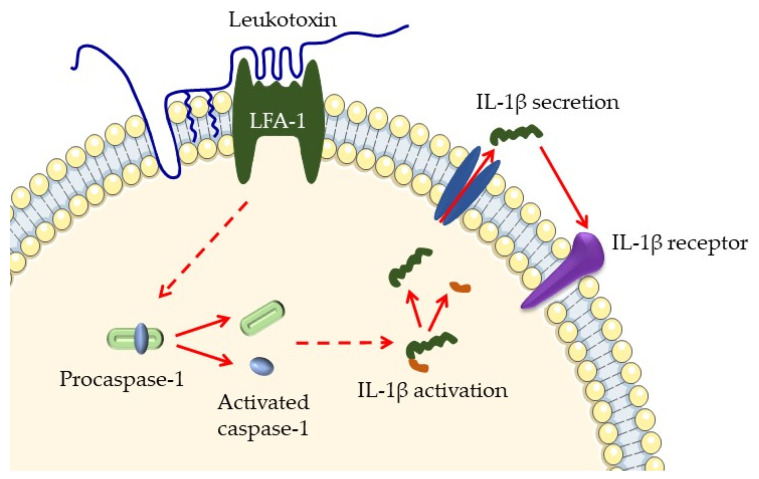
Illustration of leukotoxin interaction with leukocytes. Leukotoxin binds to the dimeric lymphocyte function-associated antigen-1 (LFA-1) on the cell membrane, where it is anchored by the incorporation of a fatty acid into the phospholipid bilayer of the membrane. Although the intracellular signaling pathways are not yet fully understood, this interaction with the target cell leads to cleavage of procaspase-1 and its activation to caspase-1, activating IL-1β, which is secreted by the cell. IL-1β is a proinflammatory cytokine that regulates the balance between catabolic and anabolic processes in tissue homeostasis. This process is of particular interest in degenerative tissue diseases such as periodontitis. It was modified according to Johansson and Kalfas [40] with a CC license.

*A. actinomycetemcomitans* release outer membrane vesicles (OMVs) and produce two exotoxins during normal growth. Leukotoxin (LtxA) is a major virulence factor that targets and kills leukocytes [8,15]. It is a 116 kDa protein produced by 56% of strains from juvenile localized periodontitis patients, and is part of the RTX family of toxins, known for creating pores in cell membranes and causing hemolysis [37]. LtxA binds to the lymphocyte function-associated antigen-1 (LFA-1) on leukocytes, leading to cell lysis and death (Figure 1). This action impairs the host’s immune response by reducing the number of functional immune cells [38]. By killing the immune cells, LtxA prevents phagocytosis of bacteria [39]. 

Cytolethal Distending Toxin (CDT) is a genotoxin that interferes with the host cell cycle, causing cell death and contributing to tissue destruction [8,41]. CDT is a tripartite toxin composed of three subunits: CdtA, CdtB, and CdtC. The active subunit, CdtB, has DNase activity and enters host cells, where it causes DNA damage. This leads to cell cycle arrest and apoptosis [42]. 

Lipopolysaccharide (LPS) constitutes a significant part of the outer membrane in Gram-negative bacteria, including *A. actinomycetemcomitans*. It acts as an endotoxin [15,37,43]. LPS interacts with the host’s immune system, specifically binding to Toll-like receptor 4 (TLR4) on immune cells, triggering a strong inflammatory response. While LPS is not a traditional toxin, its ability to induce inflammation contributes to periodontal tissue destruction and the progression of periodontal disease [37]. 

The first outer membrane proteins (Omp) associated with the rough colony variants of *A. actinomycetemcomitans* were named RcpA and RcpB (rough colony proteins A and B). These proteins were identified as being linked to the rough colony phenotype [44,45]. The genes responsible for the production of these fimbriae and associated proteins are organized within a specific region of the bacterial genome called the tad (tight adherence) locus. This locus includes 14 genes: nine *tad* genes, three *rcp* genes, and two *flp* genes. The tad macromolecular transport system, encoded by the *tad* locus, is a specialized type of type II secretion system. This system is responsible for the tight adherence of the bacterium to surfaces and the assembly of fimbriae [46]. Also involved in adhesion to epithelial cells are OmpA1 (also known as Omp29), which often plays a dual role in adhesion and interaction with the host immune system, and Omp100 (also known as ApiA), which promotes adhesion and helps the bacterium to bind to various surfaces and host tissues [8,47,48]. 

An important virulence factor is serum resistance, which occurs primarily in bacteria that enter the bloodstream and cause infection [8]. Due to their serum resistance, bacterial cells can evade innate immune defense mechanisms found in serum [49], such as antimicrobial peptides and the complement system [50]. Among other specific periodontal pathogens and *A. actinomycetemcomitans*, it is involved in complement activation via binding factor H, namely C4bp [51]. Consequently, A. actinomycetemcomitans interferes with the regulation of complement activity and evades complement-mediated killing in response to binding factor H [8]. The outer membrane protein Omp100 is essential for physical interaction with the negative regulator of the alternative complement pathway, factor H, and for complete serum resistance of some *A. actinomycetemcomitans* strains of serotype b and d [52]. Considering the evidence above, serum resistance can be considered to play an exceptional role in the virulence of *A. actinomycetemcomitans* and is a predominant feature in strains belonging to this species [8,53].

Enzymes like proteases and collagenases can be viewed as another mechanism of pathogenicity. Proteases are enzymes that degrade host proteins, facilitating tissue invasion and immune evasion. Collagenases are zinc-dependent enzymes that bacteria secrete to break down collagen fibers in their tough, triple-helical structure. These extracellular proteolytic enzymes are linked to two major periodontal pathogens: *A. actinomycetemcomitans* and *P. gingivalis*. Their collagenase activity decreases collagen density, contributing to the tissue damage seen in periodontal disease [37].

Iron acquisition is a critical process for the survival and pathogenicity of *A. actinomycetemcomitans*. Like many bacteria, it requires iron for various cellular processes, including respiration, DNA synthesis, and metabolic activities. However, iron is tightly regulated and sequestered by the host to limit bacterial growth, a defense mechanism known as “nutritional immunity.” To overcome this, *A. actinomycetemcomitans* has developed several pertinent strategies to obtain iron from the host environment [54]. One possibility is the production of siderophores and proteins that can metabolize heme and hemoglobin to release iron [55]. When *A. actinomycetemcomitans* is present in blood and heart tissue, lysis of red blood cells leads to the release of heme and hemoglobin, which is the pathway for iron acquisition [13]. It is important to note that not all strains of *A. actinomycetemcomitans* produce a functional protein that can bind hemoglobin (HgpA), which is important for the ability to utilize hemoglobin as a source of iron. Strain 652 produces it, in contrast to strain JP2 [15]. LtxA plays a significant role in iron acquisition, especially during infection [56].

### Biofilm

**Figure 2:**
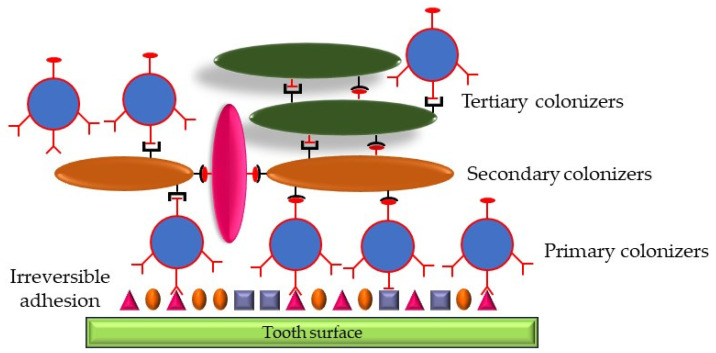
Schematic representation of dental plaque. The primary colonizers (Gram-positive species, blue circles) bind reversibly to components of the dental pellicle. Secondary colonizers (Gram-negative species, orange ovals) attach to the adhesion receptors of the primary colonizers, which leads to the maturation of dental plaque by binding Gram-negative species called tertiary colonizers (green ovals), including *Aggregatibacter actinomycetecomitans*.

Biofilm formation is an essential aspect of the pathogenicity of *A. actinomycetemcomitans*. Biofilms comprise bacteria organized into structured communities, encased in their extracellular matrix, and attached to surfaces as well as to one another. This growth mode provides several advantages to the bacteria, including protection from the host immune response and increased antibiotic resistance. Initial attachment starts with surface adhesion. The process begins with the reversible attachment of *A. actinomycetemcomitans* to a surface, such as a tooth or epithelial cell. Adhesins, fimbriae, and pili on the bacterial surface play crucial roles in this initial adherence. Specific adhesins also play a role in this. Proteins like Flp (fimbrial low-molecular-weight protein) pili, EmaA (extracellular matrix protein adhesin A), and OMPs are involved in recognizing and binding to host tissues [57,58]. *A. actinomycetemcomitans* is also identified as a tertiary colonizer (Figure 2), possessing a strong ability to bind to pre-existing bacterial colonies on tooth surfaces [57,59]. The tad locus, which includes genes for fimbriae production, is critical for biofilm formation. It encodes proteins that facilitate the tight adherence of the bacterium to surfaces and each other [60]. 

*A. actinomycetemcomitans*, being a microaerophilic organism, can thrive above and below the gingiva, adapting to both aerobic and anaerobic conditions. Since it is found in both areas, it is suggested that the supragingival plaque containing *A. actinomycetemcomitans* might serve as a reservoir, potentially leading to the spread or reinfection of this bacterium in the subgingival region [15,61]. Approximately 30% of these oral bacteria strains are resistant to benzylpenicillin. This resistance may be due to new or modified penicillin-binding proteins on the bacterial cell surface, similar to what has been observed in *H. influenzae*. Tetracyclines are commonly used alongside mechanical debridement to treat localized juvenile periodontitis. However, some studies found that 82% of 19 clinical isolates of *A. actinomycetemcomitans* were resistant to tetracyclines and possessed the *tetB* resistance gene. Additionally, the *tetB* gene could be transferred via conjugation to other *A. actinomycetemcomitans* strains and *H. influenzae*. These findings indicate that antibiotic resistance in *A. actinomycetemcomitans* is increasing and may lead to more treatment failures in the future [37]. 

Understanding these pathogenicity factors is crucial for developing targeted therapies and interventions to combat infections caused by *A. actinomycetemcomitans*.

## 4. Oncogenic Potential of *Aggregatibacter actinomycetemcomitans*

*A. actinomycetemcomitans* has several virulence factors that contribute to its oncogenic potential (Table 3). Cytolethal distending toxin is a critical factor that induces DNA damage, cell cycle arrest, and apoptosis in host cells, leading to genomic instability, a hallmark of cancer development [62,63]. Thus, *A. actinomycetemcomitans* secretes a cytolethal toxin that induces other by-products such as ROS, acetaldehyde, reactive nitrogen species, nitrosamines, and sulfides. These have oncogenic potential, manifested by mutations, DNA alkylation, and significantly impaired repair [64].

In addition, *A. actinomycetemcomitans* produces other toxins and enzymes, such as leukotoxin and collagenase, which can destroy periodontal tissues and create a favorable environment for tumor growth [11,65]. Matrix metalloproteinase-12 (MMP12), i.e., macrophage metalloelastase, plays an important role in the degradation of extracellular matrix (ECM) components [66,67]. MMP12 is involved in the pathogenesis of periodontal diseases. In addition to periodontitis, it is also present in orthodontic tooth movement (OTM), temporomandibular joint dysfunction (TMD), and oral squamous cell carcinoma (OSCC) [67,68]. MMP12 is involved in both the development and progression of tumors, focusing on cancer cell proliferation, migration, invasion, and eventual metastasis [69]. 

Recent studies have provided evidence for the direct involvement of *A. actinomycetemcomitans* in the development of oral squamous cell carcinoma (OSCC) [70]. Geng et al. demonstrated that infection with *A. actinomycetemcomitans* promotes proliferation, migration, and invasion of OSCC cells in vitro and induces the expression of proinflammatory cytokines and matrix metalloproteinases that contribute to tumor progression [71]. In contrast, Fan et al. demonstrated the association of oral pathogens, *Porphyromonas gingivalis* and *A. actinomycetemcomitans*, with a higher risk of pancreatic cancer [72]. 

The mechanisms by which *A. actinomycetemcomitans* promotes cancer development are complex [73]. In addition to the direct effects of its virulence factors, the bacterium modulates the host immune response in ways that may promote tumor growth [74]. For example, it can induce the production of immunosuppressive cytokines, such as IL-10, which can inhibit anti-tumor immunity [75]. *A. actinomycetemcomitans* also interacts with other oral bacteria, such as *Fusobacterium nucleatum*, synergistically to promote carcinogenesis [76].

The role of *A. actinomycetemcomitans* in the development of cancers outside the oral cavity has also been investigated [77]. Studies have suggested a possible link between the bacterium and the development of pancreatic cancer [72] and colorectal cancer [78]. These findings emphasize the systemic impact of the oral microbiota and the importance of considering its role in the development of various cancers [79]. The clinical potential of targeting *A. actinomycetemcomitans* in preventing and treating oral and extraoral cancers is promising [80]. Strategies to reduce bacterial load, such as antimicrobial therapy and oral hygiene measures, could potentially reduce the risk of developing cancers associated with *A. actinomycetemcomitans* [72]. 

Developing targeted therapies, such as vaccines or small molecule inhibitors against specific virulence factors of *A. actinomycetemcomitans*, such as CDT, could offer new approaches to prevent or treat these cancers [81]. Further research is needed to translate these findings into clinical practice and develop effective strategies for treating *A. actinomycetemcomitans*-associated cancers [82]. The oncogenic potential of *A. actinomycetemcomitans* highlights the need for further research to elucidate the exact mechanisms by which the bacterium contributes to carcinogenesis [83]. Understanding these mechanisms is crucial for the development of targeted strategies for the prevention and treatment of oral and extraoral cancers associated with *A. actinomycetemcomitans* [84,85]. 

## 5. Diseases Involving the Presence of *Aggregatibacter actinomycetemcomitans*

There is a growing body of evidence on the link between disruption of the oral ecosystem and systemic diseases. The association is found for systemic cardiovascular diseases, diabetes, and different malignant changes [86,87]. Pathogens and their secretions in the oral cavity activate the development of systemic diseases by stimulating the body’s immune response [30,31,88]. Periodontal pathogens as well as inflammatory mediators generated in periodontal tissues, once they enter the bloodstream, are transmitted via the blood, which in turn affects systemic health or worsens the condition of an existing systemic disease [89] (Table 4). 

It was not until 1976 that *A. actinomycetemcomitans* was associated with periodontitis in adolescents, and in 1996, it was officially established as a specific etiological agent of periodontitis, together with *Porphyromonas gingivalis* and *Tannerella forsythia* [5,91,92]. Periodontitis is one of the most common human diseases, and it is characterized by the inflammation of the periodontal soft tissues, which can lead to progressive loss of periodontal ligament, alveolar bone, tooth loss, and reduced quality of life [93]. According to different studies, it is believed that approximately 40–50% of the US population suffer from periodontitis, and 7.8% of them suffer from a severe form of the disease [94]. Patients can experience gingival inflammation (red, tender, swollen gums), bleeding after mild tissue manipulation, sensitive and loose teeth, gum recession, and bad breath (halitosis), although it can be symptomless [95]. After carefully taking the patient’s medical history, a further step in diagnosis is a clinical examination and radiographic evaluation to assess the extent of the bone loss. Currently, a classification of periodontitis in four stages is accepted, which helps in deciding about further management, rate of progression, and prognosis [96].

Periodontitis results from the interplay between specific bacterial pathogens, altered host immune response, and external risk factors such as smoking and lack of teeth hygiene habits. Periodontal microbial pathogens, especially Gram-negative anaerobic species forming biofilm, are important in sustaining chronic inflammation [96]. Today, the pathogenic role of *A. actinomycetemcomitans* in periodontitis is well established [57]. It is isolated from 50% of adult periodontitis patients and 90% of juvenile periodontitis patients [97]. A particularly strong association between specific serotype b clonal lineage (JP2 clone) and localized periodontitis is found in individuals with early-onset periodontitis. JP2 strains are highly leukotoxic, and it is assumed that the high prevalence of this clone may be the reason for the high prevalence of periodontitis in specific populations [98]. In one study that reviewed the role of *A. actinomycetemcomitans* in periodontitis, they differentiated four steps in the pathophysiological process: colonization, biofilm integration, migration below the gumline, and suppression of the mucosal host immunity. They interrogate the causative role of *A. actinomycetemcomitans* and emphasize the importance of interplay with other microbes in causing the infection, especially in cases where JP2 clones are not involved. Recently, it is more often talked about *A. actinomycetemcomitans* as a species that benefits from other microbes in biofilm, as it uses metabolic products from them but also returns the utility by promoting their overgrowth with the ability to modulate the local host defense system—leading to dysbiosis and infection [8,14,99].

Although colonization is the first step in the pathological process, *A. actinomycetemcomitans* is an integral part of normal oral flora, so that it can be present in individuals without evidence of oral cavity disease. Indeed, observational studies showed early colonization, which can be isolated from 10% of healthy children with primary dentition. This percentage increases with age, so the carrier rate among healthy adults is about 36% [97,100]. Molecular detection methods showed even higher rates of carriers, up to 100% [101]. So far, studies implicate humans as exclusive hosts for the *A. actinomycetemcomitans*. It can be transmitted vertically and horizontally between individuals, but it seems that people continue to carry the same strain throughout their lives [102,103]. Transmission primarily occurs vertically from the mother or close relatives early in life, but horizontal transmission is also possible [5]. One study showed a 14% to 60% transmission rate between couples [104]. Interestingly, even after periodontitis treatment, colonization mostly remains stable through the years [5].

With or without evident periodontal disease or dental caries, the oral cavity is the primary origin of distal body infections with an *A. actinomycetemcomitans*. Although a report of chronic wound infection with *A. actinomycetemcomitans* suggests an exogenous route of infection, there is still insufficient evidence to support this statement [105]. The prime event in the development of distal body infection with oral commensals, including *A. actinomycetemcomitans*, is the entry of bacteria into the bloodstream. It is proposed that this will happen due to a passive translocation during dental procedures, dental infections, and daily oral hygiene procedures or as a result of the actively invasive process [8]. Several virulence factors are analyzed to explain the invasive properties of *A. actinomycetemcomitans.* Still, even though studies showed the ability of *A. actinomycetemcomitans* to invade the epithelial cells in vitro, the exact mechanism of the entry into the bloodstream is not known [8,106]. For healthy immunocompetent individuals, transient bacteriemia, which happens while toothbrushing, dental flossing, or dental procedures, is generally harmless. Besides dental conditions and manipulations, other risk factors for hematogenous dissemination and distal body infections are malnutrition, diabetes mellitus, immunosuppression, aspiration, and local tissue damage [10]. But still, dental focal infections can occur in patients with no known risk factors [10,107].

Manifold distal body infections caused by *A. actinomycetemcomitans*, such as brain and mediastinum abscesses, pneumonia, arthritis, and skin infections, have been reported in the literature. However, endocarditis is by far the most commonly reported extra-oral infection [107]. Infective endocarditis (IE) is a disease with an annual incidence of up to 10 cases per 100,000 people. The incidence is increasing, especially in developed countries. It has high mortality, up to 30% in 30 days [108]. It is characterized by infection of the endocardial surface of the heart, most commonly heart valves. Staphylococci, streptococci, and enterococci are the most prevalent pathogens involved in IE [108]. Conversely, *A. actinomycetemcomitans* is not such a frequent cause of IE, and it is usually grouped with the other seven bacteria in the HACEK group. Together, they are responsible for 1–3% of all cases of IE. Among them, *A. actinomycetemcomitans* is the most common pathogen involved [109]. Although they are not pathogenetically related, they share a few significant characteristics. They are all small, slow-growing, capnophilic Gram-negative bacteria with primary oral origin [34]. The majority of patients with IE caused by *A. actinomycetemcomitans* had an underlying dental disease, but only a minority of them had dental procedures [4,109]. Other risk factors are previous heart disease (usually congenital or rheumatic) and previous valve damage or surgery [4]. Compared to other IE, *A. actinomycetemcomitans* endocarditis has a more prolonged course and more often subacute and chronic clinical picture. Patients present with intermittent fever, weight loss, anemia, and microscopic hematuria. Due to the insidious course of the disease and specific culture demands, the diagnosis of *A. actinomycetemcomitans* is usually delayed, with a mean time of 13 weeks after the onset of symptoms. Blood cultures were the primary source of diagnosis, but today, more sensitive and specific methods are recommended for the diagnosis of culture-negative endocarditis [110,111]. The second significant criterion for the diagnosis of endocarditis is vegetation, abscess, and new dehiscence of a prosthetic valve on echocardiogram or other imaging techniques [108]. Today, ampicillin in combination with gentamicin is the treatment of choice for *A. actinomycetemcomitans* endocarditis, and according to the American Heart Association, the duration of treatment should be at least four weeks for normal valve infection and six weeks for synthetic valve [112]. Surgical treatment is necessary in complicated cases of endocarditis with heart failure or valvular incompetence [34]. Finally, *A. actinomycetemcomitans* endocarditis has a relatively good prognosis with 82% survival [4]. Although results of studies regarding dental procedures as a risk factor for endocarditis are inconsistent, today, antibiotic prophylaxis is recommended for all high-risk individuals before dental extractions or manipulations of the gingival tissue, teeth, and oral mucosa [108,113].

Other *A. actinomycetemcomitans* infections can have various clinical presentations, depending on the involved body part. Symptoms are usually unspecific and most frequently reported are fever, weight loss, night sweats, and malaise. Because of this, clinicians may initially suspect malignancy or *M. tuberculosis* infection, which must be excluded [10]. 

In addition, research is underway to determine whether there is a link between periodontitis caused by *A. actinomycetemcomitans* and rheumatoid arthritis. This research also aims to clarify the potential underlying pathophysiological mechanisms [11,114,115,116]. Periodontitis supports systemic inflammation, which subsequently induces microvascular oxidative stress in the endothelium. It is described as the potential explanation for linkage with cardiovascular diseases. It is also found that *A. actinomycetemcomitans* may enhance myocardial hypertrophy through the matrix metalloproteinase-2 activation [114]. On the other hand, studies that explored the role of *A. actinomycetemcomitans* in RA suggest that it contributes to RA pathogenesis with leukotoxin A-mediated neutrophilic hypercitrullination [11,116]. Studies have also shown that leukotoxin activates the NLRP3 (NOD-like receptor family, pyrin domain-containing 3) inflammasome in macrophages, leading to the secretion of pro-inflammatory cytokines, particularly IL-1β and IL-18, which are important in both periodontitis and RA [117,118,119]. However, further research is needed to determine its exact pathogenic role and to prove a causal association. 

The main pathogenic mechanisms in diseases associated with the presence of *A. actinomycetemcomitans* are presented in Table 5.

## 6. Treatment of *Aggregatibacter actinomycetemcomitans*

The treatment of infections caused by *A. actinomycetemcomitans*, particularly those associated with periodontal disease, involves a combination of mechanical and pharmacological approaches [120]. Research has often focused on removing and extracting *A. actinomycetemcomitans*, as it has been recognized as a pathogen that does not respond well to mechanical cleaning alone [121,122]. Almost all available antimicrobial drugs have been studied for their efficacy in the treatment of periodontitis, including amoxicillin, clindamycin, doxycycline, metronidazole, tetracycline, and the mixture of metronidazole and amoxicillin [121]. The combination of amoxicillin with metronidazole is most effective when administered systemically in conjunction with mechanical debridement procedures such as scaling and root planning [120,121,123,124,125,126] to maximize the removal of bacterial biofilms. Granlund et al. investigated the antimicrobial susceptibility of strains of *A. actinomycetemcomitans* of the JP2 genotype and the non-JP2 genotype [127]. They concluded that strains of the JP2 genotype represent a subpopulation that is more sensitive to benzylpenicillin and fusidic acid than most strains of the non-JP2 genotype [127]. Low MIC (minimum inhibitory concentration) values have been reported for drugs suitable for parenteral treatment of invasive infections, such as cefotaxime, meropenem, levofloxacin, and trimethoprim-sulfamethoxazole. Amoxicillin, combined with metronidazole, is a well-established empirical antibiotic therapy for polymicrobial infections of periodontitis [127]. 

*A. actinomycetemcomitans* belongs to the HACEK group of bacteria, which is known to cause infective endocarditis [128]. Consequently, treatment can also be mechanical and pharmacological [129]. In pharmacologic therapy, ceftriaxone, a broad-spectrum cephalosporin antibiotic commonly used to treat HACEK endocarditis and usually administered intravenously, as well as other third-generation cephalosporins and quinolones are the first choice [130]. Sometimes, a combination of antibiotics, such as ampicillin and gentamicin, enhances the bactericidal effect, especially in severe cases. Ciprofloxacin is an alternative for patients who cannot tolerate beta-lactams [4,130].

Antimicrobial resistance is one of the biggest problems of our time [131,132,133]. Thus, the increasing resistance of *A. actinomycetemcomitans* to conventional antibiotics has been observed, necessitating the search for new strategies to combat this pathogen [134]. Numerous studies have looked at the anti-LtxA strategy, which is based on inhibiting the effect of LtxA. This would eliminate the colonization advantage that this toxin provides to bacteria [135,136,137]. LtxA is an ideal target to combat virulence as it is more abundant in disease-associated strains of *A. actinomycetemcomitans* [11,82]. Blocking the receptor is a relatively simple method to compete with the toxin for binding sites on the host or to bind to the toxin itself, rendering it inactive [135]. 

Due to the different types of infection and antibiotic resistance patterns, a general approach is to start with broad-spectrum antibiotics covering a range of Gram-negative bacteria, including *A. actinomycetemcomitans*, until specific culture results are available [138].

## 7. Conclusions

In conclusion, infection with *A. actinomycetemcomitans* should be considered as a possible diagnosis, given the increased likelihood of underlying periodontal disease, especially in immunocompetent patients. A link between *A. actinomycetemcomitans* periodontitis and chronic systemic diseases such as rheumatoid arthritis and cardiovascular disease has been postulated; more recent studies are attempting to prove this link and elucidate the possible pathophysiological mechanisms behind it. The oncogenic potential of *A. actinomycetemcomitans* should also be considered. Given the ability of this bacterial species to evade the host immune system and its resistance to common antimicrobial drugs, prompt and accurate diagnosis followed by targeted treatment is pivotal. Furthermore, regular dental check-ups and maintaining oral hygiene can play a significant role in preventing infections and minimizing the risk of systemic complications. Ongoing research into the virulence factors of *A. actinomycetemcomitans* and its interactions with other oral pathogens will be essential in developing more effective therapeutic strategies and preventive measures.

## Figures and Tables

**Table 1:** Scientific classification *Aggregatibacter actinomycetemcomitans*.

*Aggregatibacter actinomycetemcomitans*	
Domain:	Bacteria
Phylum:	Pseudomonadota
Class:	Gammaproteobacteria
Order:	Pasteurellales
Family:	*Pasteurellaceae*
Genus:	*Aggregatibacter*
Species:	*A. actinomycetemcomitans*

**Table 2:** Virulence and pathogenicity factors of *Aggregatibacter actinomycetecomitans*.

Virulence Factor	Main Feature or Mechanism of Action
Adhesins	adhere to host cells and surfaces
Leukotoxin (LtxA)	leukocytes lysis and death
Cytolethal Distending Toxin (CDT)	disrupts the cell cycle, causing cell death
Lipopolysaccharide	endotoxin which induces inflammation
Outer membrane proteins (Omp)	adhesion of the bacterium to surfaces and the assembly of fimbriae
Biofilm formation	antibiotic resistance
Serum resistance	avoiding the innate defense mechanisms of the immune system found in the serum
Proteases and collagenases	host proteins degradation, tissue invasion, and immune evasion facilitation
Iron acquisition	lysis of red blood cells and utilization of hemoglobin

**Table 3:** Key factors and mechanisms of the oncogenic potential of *A. actinomycetecomitans*.

Factor	Mechanism of Action	Cancer Type
Cytolethal Distending Toxin (CDT)	DNA damage, cell cycle arrest, and host cell apoptosis → ROS, reactive nitrogen species → genomic instability	Oral squamous cell carcinoma
Collagenase—matrix metalloproteinase	degradation of the extracellular matrix; proliferation, migration, and invasion of cancer cells; and metastasis	Oral squamous cell carcinoma
Leukotoxin (LtxA)	modulation of host immune response	Oral squamous cell carcinoma
Cytotoxin-associated gene E (CagE)	regulates DNA methylation	Pancreatic cancer

**Table 4:** Pathways linking oral infection and systemic responses to oral infection.

No.	Pathway	Reference
1.	metastatic inflammation caused by immune damage to oral microorganisms	[90]
2.	metastatic transmission of oral infection from the oral cavity following the development of bacteremia	[90]
3.	metastatic damage caused by the ingestion of oral microbial toxins	[90]

**Table 5:** The mechanisms of pathogenesis in diseases involving the presence of *A. actinomycetecomitans*.

Disease	Mechanism of Pathogenesis
Periodontitis	altered host immune response
Endocarditis	infection of heart valves
Rheumatoid arthritis	leukotoxin A-mediated neutrophilic hypercitrullination

## Data Availability

No applicable.
